# The Role of Regulatory Mechanisms and Environmental Parameters in Staphylococcal Food Poisoning and Resulting Challenges to Risk Assessment

**DOI:** 10.3389/fmicb.2019.01307

**Published:** 2019-06-12

**Authors:** Nikoleta Zeaki, Sophia Johler, Panagiotis N. Skandamis, Jenny Schelin

**Affiliations:** ^1^Division of Applied Microbiology, Department of Chemistry, Lund University, Lund, Sweden; ^2^Institute for Food Safety and Hygiene, University of Zurich, Zurich, Switzerland; ^3^Laboratory of Food Quality Control and Hygiene, Department of Food Science and Human Nutrition, Agricultural University of Athens, Athens, Greece

**Keywords:** staphylococcal food poisoning, enterotoxins, SEA, regulatory mechanisms, environmental factors, food supply chain, predictive modeling, QMRA

## Abstract

Prevention, prediction, control, and handling of bacterial foodborne diseases – an ongoing, serious, and costly concern worldwide – are continually facing a wide array of difficulties. Not the least due to that food matrices, highly variable and complex, can impact virulence expression in diverse and unpredictable ways. This review aims to present a comprehensive overview of challenges related to the presence of enterotoxigenic *Staphylococcus aureus* in the food production chain. It focuses on characteristics, expression, and regulation of the highly stable staphylococcal enterotoxins and in particular staphylococcal enterotoxin A (SEA). Together with the robustness of the pathogen under diverse environmental conditions and the range of possible entry routes into the food chain, this poses some of the biggest challenges in the control of SFP. Furthermore, the emergence of new enterotoxins, found to be connected with SFP, brings new questions around their regulatory mechanisms and expression in different food environments. The appearance of increasing amounts of antibiotic resistant strains found in food is also highlighted. Finally, potentials and limitations of implementing existing risk assessment models are discussed. Various quantitative microbial risk assessment approaches have attempted to quantify the growth of the bacterium and production of disease causing levels of toxin under various food chain and domestic food handling scenarios. This requires employment of predictive modeling tools, quantifying the spatiotemporal population dynamics of *S. aureus* in response to intrinsic and extrinsic food properties. In this context, the armory of predictive modeling employs both kinetic and probabilistic models to estimate the levels that potentiate toxin production, the time needed to reach that levels, and overall, the likelihood of toxin production. Following risk assessment, the main challenge to mitigate the risk of *S. aureus* intoxication is first to prevent growth of the organism and then to hamper the production of enterotoxins, or at least prevent the accumulation of high levels (e.g., >10–20 ng) in food. The necessity for continued studies indeed becomes apparent based on the challenges to understand, control, and predict enterotoxin production in relation to the food environment. Different types of food, preservatives, processing, and packaging conditions; regulatory networks; and different staphylococcal enterotoxin-producing *S. aureus* strains need to be further explored to obtain more complete knowledge about the virulence of this intriguing pathogen.

## Control and Management ofStaphylococcal Food Poisoning –Challenges Still SeekingSolutions

Foodborne diseases have been, and remain, a major global challenge in public health and economic development, with increasing numbers of incidents recorded in many countries worldwide. The WHO study on the global burden of foodborne disease estimates that 31 foodborne hazards cause 600 million illnesses and 420,000 deaths (WHO, 2015). Efforts are being made by all parties throughout the entire food chain to prevent, detect, and manage hazards resulting from the presence of foodborne pathogens. Steady changes in consumers’ eating patterns and preferences along with increasing health concerns and environmental awareness have led to a continuous and growing demand for, e.g., ready-to-eat meals, minimally processed foods, and local products (Lupien, 2007; Lappo et al., 2015). New challenges are thus relentlessly arising in the battle against foodborne diseases. This is particularly the case for minimally processed foods that provide favorable environments for most pathogenic bacteria, among them *Staphylococcus aureus*. Staphylococcal food poisoning (SFP) is a foodborne intoxication caused by staphylococcal enterotoxins (SEs) and not the bacteria themselves. Thus, the virulence of *S. aureus* as a foodborne pathogen depends on the amount of SEs formed.

Proof of the association of *S. aureus* with food poisoning was obtained in 1914 by Barber (1914) who demonstrated that food poisoning resulted from the consumption of unrefrigerated milk from a cow with staphylococcal mastitis. Dack et al. (1930) showed that a filterable toxin, later called enterotoxin, was the causative agent of SFP. Today, *S. aureus* is considered one of the most common pathogens causing food intoxication (Fetsch and Johler, 2018). In the United States, approximately 241,000 cases of foodborne illness were reported to have been caused by *S. aureus* between 2000 and 2008, placing the pathogen in the fifth place among those most commonly reported (Scallan et al., 2011). In the European Union (EU), SEs were reported to be the causes of 8% of foodborne outbreaks in 2016, rendering bacterial toxins the third most common causative agent of outbreaks (EFSA, 2017). In other parts of the world, such as Australia, intoxication by *S. aureus* accounted for 1% of all confirmed and suspected foodborne outbreaks between January 2000 and March 2012 (Pillsbury et al., 2013) whereas in China it was reported in 2013 that 12.5% of all foodborne bacterial outbreaks were caused by *S. aureus* (Wang et al., 2017). *S. aureus* is found on the skin and mucous membranes of humans and other warm-blooded animals, and is widespread in the environment. It can enter the food chain through various routes, the most common being via raw materials, food handlers, or poor hygiene in food processing equipment (Hennekinne et al., 2012; Fisher et al., 2018). In the case of *S. aureus*, contamination during food preparation and processing, or during post-production, is regarded as the main sources of risk (Argudín et al., 2010; Hennekinne et al., 2010; Kadariya et al., 2014; Castro et al., 2016). Ready-to-eat foods containing meat products and dressings, which are handled and consumed without further treatment, have been implicated in a number of SFP outbreaks (Table 1). In the EU summary report 2016 (EFSA, 2017) on foodborne outbreaks, the main vehicle for SFP outbreaks was reported to be “mixed foods” with 31% followed by milk and milk products with 22%, highlighting the impact of post-processing of foods on the development of the disease.

**Table 1:** Examples of major SFP outbreaks.

Implicated food	*n* cases	Country	Year	References
Raw milk cheese	200	United States	1958	Johnson et al. (1990)
Chicken salad	1300	United States	1968	CDC (1968)
Sausage rolls, ham sandwiches	100	United Kingdom	1971	Morris et al. (1972)
Ham	197	Flight from Japan to Denmark	1975	Eisenberg et al. (1975)
Dessert cream pastry	215	Caribbean cruise ship	1983	CDC (1983)
Cheese (sheep’s milk)	27	Scotland	1984	Bone et al. (1989)
Dried lasagna	50	France, United Kingdom, Italy, Luxembourg	1985	Woolaway et al. (1986)
2% chocolate milk	>1000	United States	1985	Evenson et al. (1988)
Canned mushrooms	102	United States	1989	CDC (1989)
Eclairs	485	Thailand	1990	Thaikruea et al. (1995)
Precooked ham	18	United States	1997	CDC (1997)
Chicken, roast beef, rice, and beans	4000	Brazil	1998	Do Carmo et al. (2004)
Low-fat milk	13,420	Japan	2000	Asao et al. (2003)
Cheese (sheep’s milk)	104	France	2002	Kérouanton et al. (2007)
Potato snack	100	India	2005	Nema et al. (2007)
Coconut pearls (Chinese dessert)	17	Île-de-France area, France	2006	Hennekinne et al. (2009)
Milk, cacao milk, vanilla milk	166	Austria	2007	Schmid et al. (2009)
Crepes	75	Japan	2009	Kitamoto et al. (2009)
Raw milk cheese	23	France	2009	Ostyn et al. (2010)
Raw milk cheese	14	Switzerland	2014	Johler et al. (2015)

*Staphylococcus aureus* is able to grow and produce enterotoxins under a wide range of temperatures, pH values, water activity (*a*_w_) levels, and sodium chloride concentrations (Le Loir et al., 2003; Paulin et al., 2012; Adams et al., 2016). This robustness of the pathogen in a wide range of environmental conditions broadens the diversity of foods in which *S. aureus* can grow and express virulence (Hennekinne et al., 2012; EFSA, 2017). Thus, any food product that can support the growth of *S. aureus* could pose a risk in terms of SFP. Additional post-production handling of the food could further increase the risk of disease due to *S. aureus* contamination. Identifying the risks associated with *S. aureus* in the food handling and production environment, such as breach of hygiene, is one of the most important factors in the control of SFP. However, the dissemination of the pathogen in nature, its robustness under diverse conditions, and the level of knowledge of people involved in food processing are only some of the challenges associated with this pathogen. To these must be added specific differences among *S. aureus* strains including the variation in SE expression and production levels, the different types of mobile genetic elements encoding the *se* genes, and the accompanying range of different regulatory mechanisms. As stated above, SFP leads to intoxication due to the ingestion of enterotoxins preformed in the food (Fetsch and Johler, 2018). SEs produced by *S. aureus* are resistant to most of the treatments that eliminate bacterial cells, such as heat treatment and low pH, and can therefore be present in food even after processing. Their resistance to proteolytic enzymes also enables them to pass through the digestive tract unaffected, thus increasing the risk of illness (Le Loir et al., 2003). The key element in preventing SFP outbreaks is thus to control the production of enterotoxins.

Among the SEs and SE-like proteins produced by *S. aureus*, those usually associated with SFP are the five classical SEs: SEA–SEE (Wieneke et al., 1993; Cha et al., 2006; Kérouanton et al., 2007; Rajkovic, 2012). Staphylococcal enterotoxin A (SEA) is most frequently reported as the causative agent of SFP outbreaks (80% of cases), followed by SEB, SEC, and SED (Pinchuk et al., 2010; Hennekinne et al., 2012; Sospedra et al., 2013). The challenge in understanding and controlling the production of SEs lies in the complexity and diversity of the encoding genetic elements and regulation mechanisms. The *se* genes are carried on various genetic elements such as plasmids, bacteriophages, pathogenicity islands (SaPIs), and the enterotoxin gene cluster (*egc*) (Genigeorgis, 1989; Le Loir et al., 2003; Argudín et al., 2010; Hennekinne et al., 2012). Different regulatory mechanisms are involved in SE expression depending on the genetic element carrying the SE.

**FIGURE 1:**
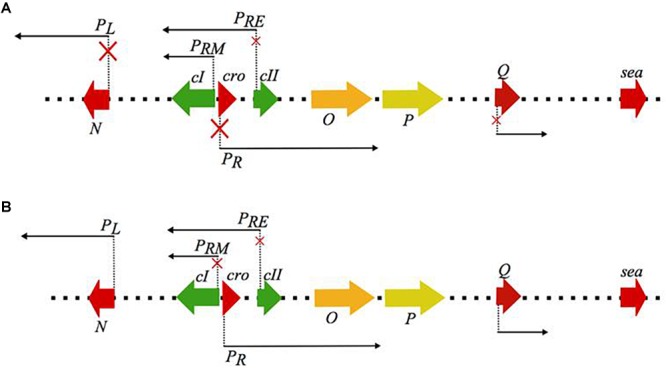
Schematic representation of the regulatory events occurring during **(A)** lysogenic and **(B)** lytic mode of the λ phage life cycle, that serves as the model for the closely related *Siphoviridae* genera. The red **×** on the promoter arrows indicate repression of transcription from the respective promoters. During the lysogenic mode *cI* autoregulates its expression through the P_RM_ promoter while it represses the lytic promoters P_R_ and P_L_ which regulate the early (N, O, P) and the late lytic genes located downstream the Q promoter, including the virulence genes, like the *sea* gene. Under the events that favor the lytic mode, *cI* autoregulation from P_RM_ seizes and transcription from the lytic promoters is initiated. The regulatory protein of the lytic mode is *cro*, which represses the expression of *cII* and therefore re-establishment of the *cI* expression (figure modified from Oppenheim et al., 2005).

The mechanisms controlling SE production in *S. aureus* are numerous and include *se* gene promoter regions, multiple global regulators of virulence, such as the accessory gene regulator (*agr*), the staphylococcal accessory regulator (*sar*), the repressor of toxins (*rot*), the *sigB* factor, and the two-component system *S. aureus* exoprotein expression (*sae*), in addition to a cytoplasmic SEB form that controls SEB production (Gustafson and Wilkinson, 2005). Selected classical enterotoxins with respective regulatory mechanism/s/ are summarized in Table 2 and also well accompanied in Figure 1 in the review by Fisher et al. (2018) that gives an overview of the relationship between growth environment/condition, regulatory mechanisms, and type of SE (Fisher et al., 2018). Knowledge concerning the regulatory mechanism of a SE can therefore be of great value in the effective prevention of *S. aureus* intoxication, as the appropriate interventions may differ depending on the SE-producing *S. aureus* strain. SEA and SED can be used to exemplify different mobile elements and different regulatory systems, as SEA is encoded by a bacteriophage, while SED is encoded by a plasmid. The latter is partly regulated by the *agr* system, a quorum sensing system that enables *S. aureus* to respond to cell density, and SED is thus mainly produced during the transition from the exponential to the stationary growth phase of the microorganism (Schelin et al., 2011). The regulation of SEA, however, has been linked to the life cycle of the bacteriophages carrying the *sea* gene, with SEA production being the highest during the exponential growth phase, at the peak of replication (Derzelle et al., 2009; Schelin et al., 2011). Furthermore, these regulatory mechanisms are affected in different ways by the environmental conditions in a food product, such as salt content, water activity, and pH (Schelin et al., 2017). This can have a further impact on the production of the respective enterotoxins, making them difficult to control.

**Table 2:** Selected *S. aureus* enterotoxins regulated by Agr, SarA, σ^B^, Rot, and SaeRS.

Enterotoxins	Agr	SarA	σ^B^	Rot	SaeRS	References
SEA	0	nd	0	0/-	nd	Tremaine et al. (1993), Tseng et al. (2004), Kusch et al. (2011), Sato’o et al. (2015)
SEB	+/0	+	–	–	+	Compagnone-Post et al. (1991), Regassa et al. (1991), Schmidt et al. (2004), Tseng and Stewart (2005), Kusch et al. (2011)
SEC	+	+	+	nd	nd	Chien et al. (1999), Regassa et al. (1991), Voyich et al. (2009)
SED	+/0	+	–	–	nd	Bayles and Iandolo (1989), Tseng et al. (2004), Sihto et al. (2015, 2016a)
SEE	nd	nd	nd	nd	nd	

*+, activation; -, repression; 0, no effect; nd, not determined.*

The location of the *se* genes on mobile genetic elements presents an additional challenge in SFP control, as it supports horizontal gene transfer between different *S. aureus* strains, and therefore the dissemination of *se* genes (Hennekinne et al., 2012). The transfer of genetic elements in *S. aureus* has contributed to strain variability and enhanced virulence, as *S. aureus* strains usually carry more than one *se* gene (Becker et al., 2003). These evolutionary trends in *S. aureus* call for constant research to identify potentially new strains and to understand their behavior and virulence expression with regard to SFP.

## A Glimpse of *S. Aureus* Entry Routes Into the Food Chain

The diversity of food products implicated in SFP is apparent from documented outbreaks (Table 1). The robustness of *S. aureus* under a wide range of environmental conditions should be considered a virulence parameter, making it difficult to predict the general behavior of the pathogen in various food matrices under different environmental conditions. The series of events in an SFP outbreak typically include the following: (i) the presence of the pathogen in raw materials or the food handler(s), (ii) contamination of the food, for example, through processing equipment or through the food preparer, (iii) inappropriate storage conditions and/or inadequate temperature control that allow for bacterial growth and enterotoxin production, and (iv) ingestion of contaminated food containing a sufficient amount of SE to trigger symptoms of the disease. The majority of well-documented outbreaks indicate that the most common means of contamination in cases of SFP are poor hygiene practices during the processing, cooking, and distribution of food products. Inadequate cooling of food products is the main cause of *S. aureus* growth leading to disease (Pereira et al., 1996; Asao et al., 2003; Schmid et al., 2009; Hennekinne et al., 2012).

One of the first well-documented SFP outbreaks was reported by Denison (1936) and involved the consumption of contaminated cream puffs by high school students. In this case, the source of contamination was not actually identified. In more recent years, a large-scale SFP outbreak affected as many as 13,420 individuals, and involved the consumption of dairy products that contained SEA (Asao et al., 2003). From the investigation of this outbreak it was found that the source of contamination was the powdered skim milk used to produce the dairy products involved in the outbreak. The most interesting factor in this case was that the contaminated products had been heat-treated, in some cases as much as three times, at 130°C for 2 or 4 s, and bacterial cells of *S. aureus* were completely eliminated. SEA retained, however, both its immunological and emetic activity and led to food poisoning (Asao et al., 2003). Besides the classical types of food products associated with *S. aureus* contamination an example of a more recent study investigated the prevalence on various retail vegetables in China between 2011 and 2016 (Wu et al., 2018). Although the levels of *S. aureus* were moderate, it was found in 5.73% of the samples and lettuce was the most common vegetable. In addition to the conventional entry routes connected with production and processing environments, the global trade and especially the illegal transportation of food across borders have also been found to contribute to the transmission of *S. aureus* (Müller et al., 2016; Rodriguez-Lazaro et al., 2017). Another alarming concern is that not only enterotoxigenic but also methicillin-resistant *S. aureus* (MRSA) strains are increasingly being isolated from a variety of meat and dairy-based products as well as ready-to-eat foods indicating that the food chain is one additional and alternative reservoir as well as transmission route for antimicrobial resistant bacteria (Sergelidis and Angelidis, 2017; Islam et al., 2019; Wu et al., 2019).

Staphylococcal enterotoxin A is one of the more extensively studied SEs, mainly due to the fact that it accounts for 80% of reported SFP cases. Its worldwide predominance has been extensively documented (Argudín et al., 2010). In a study of 359 SFP outbreaks in the United Kingdom that took place between 1969 and 1990, it was shown that 79% of the *S. aureus* strains involved produced SEA (Wieneke et al., 1993). SEA was the only enterotoxin detected in 56.9% of the outbreaks, while in a few cases it was detected together with SED, SEB, and SEC, or with SEB and SED. The sources implicated included meat, poultry, and products thereof, especially ham and chicken. The *sea* gene was also the predominant enterotoxin gene detected in dairy products implicated in SFP outbreaks in Brazil (Veras et al., 2008). SEA was the most frequent causative agent in 69.7% of SFP outbreaks in France between 1981 and 2002 (Kérouanton et al., 2007). A challenging aspect of this high prevalence with regard to controlling SFP intoxication is that SEA is one of the few SEs whose regulation is not dependent on the *agr* system. A number of studies have investigated the expression of the *sea* gene in connection with alternative regulatory mechanisms, in order to understand the conditions under which the enterotoxin is produced. In the following section, SEA will be used as a characteristic example of the challenges from different genetic backbones; the regulatory mechanisms; and environmental stress responses to the robustness, growth behavior, and strain variations associated with SE production by *S. aureus* leading to SFP.

## Expression and Production of Enterotoxins in Food

### Staphylococcal Enterotoxin A

The *sea* gene encoding for SEA is located on the genome of a polymorphic family of lysogenic bacteriophages, the *Siphoviridae* family. Localization of the *sea* gene on these phages has been found to affect its expression and the production of SEA, creating variations between the SEA-producing strains and the virulence these can express when found in food (Betley and Mekalanos, 1985; Borst and Betley, 1994; Zeaki et al., 2015a).

To date, five *S. aureus* bacteriophages are known to carry the *sea* gene, namely: ΦSa3ms, ΦSa3mw, Φ252B, ΦNM3, and ΦMU50A (Goerke et al., 2009; Deghorain and Van Melderen, 2012). These are temperate bacteriophages and are thus able to establish a permanent symbiosis with their bacterial host, known as lysogeny. Lysogenic bacteria (or “lysogens”) are cells where the phage genome has integrated into the bacterial chromosome and will be steadily transmitted to the bacterial progeny as a prophage (Thieffry and Thomas, 1995). In this state, the lysogenic bacterium will inherit the characteristics attributed to phage genes, such as resistance to antimicrobial substances and virulence expression.

In addition to inheriting phage genes, the symbiosis of *S. aureus* with the *sea*-carrying phages leads to another challenge, that of strain variation regarding virulence. It has been established, through a number of studies, that the SEA-producing *S. aureus* strains can be categorized into high and low SEA producers, depending on the *sea*-carrying prophage they harbor (Borst and Betley, 1994; Wallin-Carlquist et al., 2010a; Cao et al., 2012). Wallin-Carlquist et al. (2010a) showed that there are two *sea* variants: *sea_1_* and *sea_2_*; the high-SEA-producing strains carried *sea_1_*, while *sea_2_* was found in the low-SEA-producing strains. It could therefore be proposed that the SEA-producing strains bearing the *sea_1_* gene variant are more likely to cause SFP than those bearing the *sea_2_* gene variant.

**FIGURE 2:**
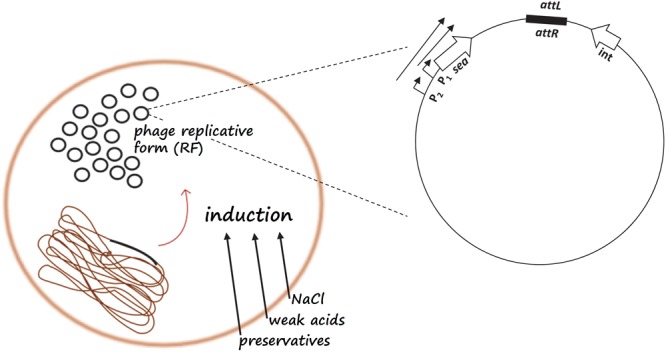
Schematic representation of the *sea* gene regulatory mechanism. Food parameters such as NaCl, weak acids, and preservatives may lead to prophage induction and replication of the circular, replicative form (RF) of the phage genome, resulting in an increase in RF copies in the cell. Prophage induction will initiate transcription from the latent promoter P_2_ resulting in the production of a longer *sea* transcript in addition to the *sea* transcript from the endogenous P_1_ promoter.

The state of lysogeny, although very stable, can be disrupted by certain environmental conditions (i.e., the presence of weak acids, high NaCl concentration, UV irradiation, and DNA damage by chemical agents), and in such cases, the lytic response is initiated by the phage; a process known as prophage induction (Figure 1; Oppenheim et al., 2005). Prophage induction has been found to increase the amount of SEA produced by some SEA-producing strains, and thus increases the probability of SFP (Figure 2).

In 1994, Borst and Betley identified an endogenous promoter responsible for *sea* mRNA expression, immediately upstream of the gene. Cao et al. (2012) demonstrated the differences between the designated groups of high- and low-SEA-producing strains regarding the expression of *sea* mRNA. Specifically, they showed that relative quantification of *sea* mRNA originating from the endogenous *sea* promoter (designated P_1_) was only possible in the high-SEA-producing strains, i.e., those bearing the *sea_1_* gene variant. Furthermore, it was shown that some of these strains had the ability to produce increased amounts of SEA when cultures were subjected to prophage induction using mitomycin C. In these strains, a second *sea* transcript was detected and quantified originating from a latent promoter (P_2_) located upstream of the endogenous *sea* promoter (P_1_) (Sumby and Waldor, 2003; Cao et al., 2012). The high-SEA-producing group was thus divided into two sub-groups, the inducible high-SEA producers, for which the expression of two *sea* transcripts and higher SEA levels was observed after induction, and the non-inducible high-SEA producers, for which no effects on *sea* transcription and translation was seen (Cao et al., 2012).

Apart from the impact on *sea* mRNA expression, prophage induction also causes changes in the number of copies of the circular, replicative form (RF) of the phage genome in the cell. At a particular point during the life cycle of the phage, the host cell cytoplasm will contain a considerably higher number of RF copies and, consequently, of phage-associated genes available for potential transcription. Therefore, in the case of the *sea*-carrying phages, prophage induction and phage genome replication increase the *sea* gene pool in the cell, and the amount of *sea* mRNA produced, possibly enhancing SEA production (Figure 2). This hypothesis was investigated in a study by Zeaki et al. (2015a), where the RF copies were quantified before and after prophage induction. From this analysis it was demonstrated that the inducible high-SEA-producing strains exhibited high numbers of RF copies after prophage induction and increased SEA levels, in contrast to the non-inducible strains.

The findings demonstrating all the dimensions through which the life cycle of the *sea*-carrying phages regulates SEA production were generated under laboratory conditions. This highly complex regulatory mechanism has an immediate impact on the virulence of *S. aureus* and SFP, since several parameters encountered in food or during food production could lead to prophage induction, and thus to high SEA levels. To elucidate the increased risk of SFP due to SEA production in the food environment, studies on food matrices and/or including food parameters, such as high NaCl concentration and/or weak acids, should be performed.

### SEA Production in Food

Food provides a rich source of nutrients for all types of bacteria. Guidelines for good manufacturing practices and controls for food safety have been developed and were adopted by the food industry to minimize the contamination of food products. Various preservation methods are employed to further prevent the growth of pathogenic bacteria due to potential post-production contamination (Adams et al., 2016). These methods make the environment less favorable for bacterial growth, by reducing the oxygen level, the pH, and water activity value. However, in the case of *S. aureus* and SEA production, adverse conditions in the food environment could lead to prophage induction and increased SEA production.

A number of studies have investigated food parameters affecting the growth of *S. aureus* and SEA production (Genigeorgis, 1989; Belay and Rasooly, 2002; Wallin-Carlquist et al., 2010a,b; Rosengren et al., 2013; Sabike et al., 2014; Zeaki et al., 2014, 2015b; Hu et al., 2018). The generally accepted limits for SE production are temperatures between 10 and 48°C, a pH range of 4–9.6, and NaCl concentrations of 0–10%. However, most studies have focused on rather extreme parameters that inhibit either the growth of the pathogen and/or SE production, rather than investigating a range typically found during food production and preservation. In addition, they largely omitted effects on a regulatory level. The impact of regulatory mechanisms on the production of SEs can be significant and can vary between *S. aureus* strains producing the same enterotoxin. In a study of Wallin-Carlquist et al. (2010a), it was shown that acetic acid increased the expression of the *sea* gene at pH 6.0, a pH level at which increased copy numbers of the gene were also detected. Zeaki et al. (2015b) observed a similar effect on the copy number of *sea* and the *sea*-carrying phage genome, when 2% NaCl was added to the growth medium. In both studies, however, the increase in expression and the number of copies of the gene was not translated into increased SEA levels. This could be attributed to inhibition of the biosynthesis of SEA or its secretion from the *S. aureus* cell due, for example, to the accumulation of compatible solutes such as proline, glycine, and betaine by the cell under osmotic stress, which are also essential for SEA synthesis. However, these amino acids could be available from alternative sources in a food environment, and thus SEA synthesis could re-establish and reach levels causing SFP. The above example highlights the importance of considering the composition of a food product when deciding on preservation treatments for the effective inhibition of SE production. It has been shown, for example, that the addition of glucose reduces enterotoxin production due to catabolic repression and as a consequence of pH reduction due to the fermentation of carbohydrates (Jarvis et al., 1975; Smith et al., 1986; Hallis et al., 1991; Weinrick et al., 2004).

Wallin-Carlquist et al. (2010b) investigated the growth of *S. aureus* and the production of SEA in four different processed pork products (boiled ham, hot-smoked ham, Serrano ham, and black pepper salami). The different food environments were found to have different effects. Cell growth and SEA production at levels likely to cause SFP were observed in the boiled and hot-smoked ham early in the incubation period, while in the harsher environments (i.e., high salt and fat content, low water activity, low pH, presence of competing microbiota, and immobilized growth) of Serrano ham and black pepper salami, growth and SEA production were observed after 5 days of incubation in the Serrano ham, and not at all in the black pepper salami. Interestingly, *sea* expression was detected throughout the incubation period, in contrast to what has been observed in broth, in which *sea* expression peaks at the transition from the exponential to the stationary growth phase, and then declines (Borst and Betley, 1993; Derzelle et al., 2009; Wallin-Carlquist et al., 2010a,b). These observations evidence the variability of SE expression depending on the product of interest, and the differences in *sea* gene regulation between food matrices and laboratory conditions.

In a more recent study by Zeaki et al. (2014) on pork sausages, the inter-strain variability of *S. aureus* with respect to growth, *sea* expression, and SEA production was investigated. The pork sausages were inoculated with three *S. aureus* strains, previously known to produce different amounts of SEA when grown under the effect of mitomycin C, despite their similar growth patterns. The temperature (15°C) used during filling of the meat paste into the skin in sausage production was used for incubation, as this also represents an inadequate refrigeration temperature. It was found that although all three strains exhibited very similar growth patterns during the 14 days of incubation, the levels of SEA produced differed considerably. Two of the strains previously characterized as high-SEA producers produced SEA levels in the pork sausage environment high enough to cause food intoxication, while the low-SEA-producing strain did not. In addition, one of the two high-SEA producers yielded significantly higher SEA levels than the other high-SEA-producing strain. These two strains had previously been found to produce similar levels of SEA in a laboratory experiment in broth. This observation is related to prophage induction and indicates that some food parameters may trigger this process, increasing *S. aureus* virulence.

The importance of the findings presented above becomes clear when considering the methods used to ensure the safety of a food product for consumption. The guidelines for microbial safety of food products are based on acceptable pathogen counts in a critical food portion (ECDC, 2005). In the guidelines in the US Food and Drug Administration’s “Bad Bug Book,” the level of *S. aureus* producing enterotoxins that could cause intoxication is defined as ≈10^5^ cfu/g. Thus, SFP risk assessment is based on the level of *S. aureus* detected in the final product. Yet, as was shown in the study by Zeaki et al. (2014), SEA production varied between the studied strains, despite the fact that the growth patterns of the microorganisms were similar. Strain-specific variation has furthermore been demonstrated in several other studies in regard to different toxins and regulatory mechanisms (Blevins et al., 2002; Cassat et al., 2006; Sihto et al., 2016b; Susilo et al., 2017). Therefore, current methods of evaluating the safety of food products with regard to *S. aureus* are inadequate and should be complemented with more information regarding the virulence and diversity of individual strains and SEs.

### Other Classical Enterotoxins

Staphylococcal enterotoxin A presents a distinctive complexity in its regulatory system compared to the majority of SEs, in addition to being the one most often implicated in SFP outbreaks. Nevertheless, SEs such as SEB, SEC, and SED have been recorded as sources of SFP cases, with SEB being the second most common cause after SEA (10%) (Pinchuk et al., 2010). Therefore, a deeper understanding of their behavior when found in food is essential. In fact, comparative knowledge of the regulatory mechanisms governing the expression and production of SFP-relevant SEs would be of great value, for example, for risk assessment models on *S. aureus* virulence.

The response of *S. aureus* to environmental changes is controlled by a highly complex network of intertwined regulatory pathways, including quorum-sensing and other two-component systems as well as trans-acting regulatory proteins (Fisher et al., 2018; Poupel et al., 2018). SEB, SEC, and SED, in contrast to SEA, are regulated by the *agr* quorum-sensing system to different extents (Table 2). The latter has been extensively studied under laboratory conditions regarding its up- and down-regulation by parameters such us sodium chloride, glucose, weak organic acids, and other. Several trans-activating regulators such as σ^B^, SrrAB, or the Sar family of regulatory proteins also influence *agr* expression (Fisher et al., 2018). Some studies have further looked into the impact of the up- and down-regulation of *agr* on the production of SEs. For example, in the study of Regassa et al. (1991), the effects of glucose and pH on the expression of *agr* and *sec* were evaluated. It was revealed that *agr* expression decreased dramatically under the presence of glucose and when the pH was 5.5. Similarly, glucose reduced the levels of *sec* mRNA and its impact was further enhanced when pH was 5.5 or non-maintained. In another study, on SED expression under glucose (30%) and lactic acid stress (pH 6.0), it was shown that glucose stress decreased the expression of SED, while lactic acid stress had no significant impact (Sihto et al., 2016b). When six different organic acids were investigated on their impact on *S. aureus* growth (strains FRI-100, S6, FRI-137, and FRI-472) and SE (SEA, SEB, SEC, and SED) production, it was revealed that their effect varied between strains and type of SE. For example, SEA was decreased in the presence of most acids. However when *S. aureus* FRI-100 was grown with pyruvic or propionic acid, its concentration increased. SEB was the enterotoxin the least affected by the presence of all acids. In addition, for strain S6, though its growth was the most inhibited, SEB levels were distinctly high with all acids investigated, higher than what could be expected when considering growth (Domenech et al., 1992).

The presented examples provide useful information for the behavior of *S. aureus* when grown under the effect of compounds used in food production. However, little knowledge exists, also in the case of the *agr*-regulated SEs, on their response in food. Moreover, the level to which *agr* regulates the production of these SEs has not been completely identified and other potential mechanisms affecting these SEs are yet to be discovered.

The existing food studies provide interesting observations on the impact of the food environment on SE production. In a study by Alibayov et al. (2015) on SEC expression and production in four different meat products (chicken ham, pork ham, pepper beef salami, and turkey ham), significant differences were observed on *sec* expression among these products, though growth followed a similar pattern. The differences were attributed both to the differences in fat content between the products, as well as the harshness of each of them (NaCl concentration and pH). When the effect of NaCl on *sed* expression was investigated by Sihto et al. (2015), a decrease was observed in the expression of the gene in the studied strains. One *S. aureus* strain, however, exhibited a trend toward increased *sed* expression, suggesting possible induction of *sed* expression by NaCl stress. Briefly, studies in milk on SEC have demonstrated substantial reduction of both expression and production of the enterotoxin (Valihrach et al., 2013). In the same study, differences were observed between the expression profiles of *sec* and other *se* genes under the same conditions.

### Newly Described Enterotoxins

While the role of newly described SEs in SFP has been controversially discussed, evidence is strongly suggesting a contribution to SFP (for a comprehensive review, see Fisher et al., 2018). Still, data on the expression of newer SEs and regulatory elements involved are scarce and expression in the food matrix is largely unknown. Transcription of the phage-encoded newer enterotoxins *sek* and *seq* is linked to the phage’s life cycle and can be induced by mitomycin C (Sumby and Waldor, 2003). Kusch et al. (2011) showed that transcription of *sek* and *seq* is not affected by loss of SaeS and σ^B^ in the strains COL and MA19, whereas expression of the *egc* encoded newer enterotoxins (*seg, sei, sem, sen, seo, seu*) depends of σ^B^. Expression of the transposon-associated *seh* has been suggested to be Agr independent (Lis et al., 2012) and controlled by Rot, SaeR, and SarR homologs (Sato’o et al., 2015) as well as σ^B^ (Kusch et al., 2011). Sato’o et al. (2015) suggested that Rot binds directly to the *seh* promoter, thus leading to *seh* mRNA transcription. They also reported that when comparing SE production in laboratory media and meat product using ELISA, many SEs could only be detected in laboratory medium and not in the food matrix, including SEB, SEC, SED, and the newer enterotoxins SEG and SEI (Sato’o et al., 2015). Expression of *selj* was suggested to be Agr-independent (Zhang et al., 1998). A recent study by Schubert et al. (2017) showed that the production of SER in meat juice exceeded SER production of the same strains in milk by a factor of between 15 and 269. Their findings also show pronounced strain-specific variation in *ser* expression.

## Challenges and PossibilitiesUsing Risk Assessment to PredictEnterotoxin Production andStaphylococcal Food Poisoning

Describing and quantifying bacterial growth in food environments are rather complex – as complex is the response of the bacteria in the food matrix itself. Furthermore, changes in food production methods and pathogen evolution create a need for continuous research on microbial risk assessment. Understanding the genetic mechanisms that regulate the phenotypic responses of bacteria, and incorporating this knowledge into existing predictive models, will greatly improve hazard identification and pathogen control. Risk assessment associated with SFP has another degree of complexity due to the necessity of assessing SE production, and not just the presence or absence of the organism. This means that the strain of *S. aureus*, the type and amount of SE produced, and the possible correlation between growth and SE production must be evaluated. The potential for intoxicating amounts of SE to be present in a food product, even in the absence of viable *S. aureus* cells, should also be considered when assessing the risk posed by this organism (Cretenet et al., 2011).

A number of studies have been carried out, with focus on assessment of *S. aureus* risk of growth and toxin production in various products, including milk, unripened raw-milk cheese, home-cooked foods, and cream-filled bakery products (Lindqvist et al., 2002; Stewart et al., 2003; Taulo et al., 2008; Heidinger et al., 2009; Kim et al., 2009). As one of the major components of risk assessment is the exposure assessment, the use of predictive models is an essential tool for estimating the spatiotemporal changes of *S. aureus* population in the food chain, the attainment of toxin producing levels in target foods, and the amounts of toxins present in foods at the time of consumption. The existing predictive models are capable of simulating dynamics of cell populations and toxin production over time, in response to the main factors, controlling microbial growth, such as temperature, pH, and water activity. Both kinetic and probabilistic models are available, as detailed in the following lines, some also being readily available in predictive modeling databases, such as ComBase. Nonetheless, the polynomial models of ComBase are generic models trained on responses of multiple *S. aureus* strains to temperature (7.5–30°C), pH (4.4–7.0), and *a*_w_ (0.907–1) in broth. As a counteract to the limitations of broth-based models, growth simulations via the above predictive modeling platforms enable the users to assess the impact of variability in population dynamics, thereby assessing the stochastic response of microorganisms, which is an important feature for exposure assessment.

Regarding the food-specific models, Fujikawa and Morozumi (2006) developed a model describing the rate of SEA production on the basis that the enterotoxin is formed when *S. aureus* levels are greater than 6.5 log cfu ml^-1^, and that the relationship between SEA production and growth is linear in the temperature range 15–32°C. In a study by Valero et al. (2009), the influence of temperature, pH, and water activity on the growth of *S. aureus* was evaluated and fitted in a growth/no growth model. Lindqvist et al. (2002) used predictive modeling and survey data in combination with probabilistic modeling to simulate the levels of *S. aureus* at the time of consumption of unripened cheese made from raw milk. In this way, they evaluated the risk associated with consumption of this cheese and found that the initial *S. aureus* population, pH, and storage time were the main risk factors. Barker and Goméz-Tomé (2013) took risk assessment one step further and developed a probabilistic model for the representation of the risks that arise in pasteurized milk from the presence of *S. aureus*, and particularly SEs, during the entire production chain. Specifically, they implemented probabilistic analysis with a Bayesian belief network, and in that way introduced the concept of biotraceability. Thus, the model developed by Barker and Goméz-Tomé (2013) allowed the identification of the main hazard sources. This in turn allowed for conclusions on where pre-emptive action should be taken in the milk production process to increase its efficiency and safety (Barker and Goméz-Tomé, 2013).

A limitation of the models developed to date, such as those described above, is the fact that the risk is assessed based on predicted levels of *S. aureus* that have been associated with enterotoxin production, rather than predicted enterotoxin levels in the final product, or the actual dose–response. Moreover, the information usually included in these models is generic, i.e., it does not distinguish between the different types of enterotoxins and their gene regulatory mechanisms. The latter is a key challenge for assessing the severity of the disease and for characterizing the impact of the food (micro)-environment and storage conditions on the ability of *S. aureus* to produce toxin. In this context, the marginal conditions allowing production of SEA are different from those that permit growth of *S. aureus* (Borneman et al., 2009; Ding et al., 2016), whereas toxin production may also be induced by severe stresses (Cao et al., 2012). Nonetheless, despite the existing preliminary indications, whether and how frequently these types of stress are associated with food environments requires further investigation (Zeaki et al., 2014, 2015b). Overall, the aforementioned evidence suggests that the toxin producing *S. aureus* levels are practically variable and a generic threshold cannot be reliably set. Usually in quantitative microbial risk assessment (QMRA) approaches, the prediction of *S. aureus* growth by the time of consumption is linked to the correlation between levels of *S. aureus* and toxin and the assumption of a minimum toxin amount of 20–50 ng for the occurrence of intoxication symptoms, in order to assess the risk of intoxication (Kim et al., 2009).

Many of the existing studies highlight the need for more information regarding the dynamics of *S. aureus* growth in relation to gene expression and enterotoxin production, the impact of different food environments on gene regulation, and relevant dose–response data, to improve risk assessment (Lindqvist et al., 2002; Fujikawa and Morozumi, 2006; Schelin et al., 2011; Barker and Goméz-Tomé, 2013). Rosengren et al. (2010) addressed the knowledge gaps in risk assessment associated with *S. aureus* in fresh and short-time ripened cheeses in Sweden. The areas where gaps were identified included producers’ practices at farm dairies, the properties and the virulence of various *S. aureus* strains, the sources of *S. aureus*, and the impact of pasteurization and starter cultures on *S. aureus* levels. Given that *S. aureus* is considered a rather “poor” competitor, predictive models have also quantified the competitive effect of starter cultures (e.g., lactic acid bacteria) on the growth of *S. aureus*, in response to temperature (Le Marc et al., 2009). The model was based on the so-called “*Jameson effect*,” which practically describes the growth cessation of the weaker organism in a binary culture, when the competitor reaches a threshold population, which is higher than the other organisms.

The complexity of SE production and the network of synergistic signals that regulate enterotoxin expression highlight the need for physiology-based, or even genotype-specific *S. aureus* risk assessments, thus, paving the way of increasingly integrating -omics in QMRA. These issues are further discussed in the following lines. The *S. aureus* strain and, in the case of phage-encoded enterotoxin genes such as *sea*, the respective *sea*-carrying phage are critical parameters influencing the relevance of *S. aureus* as a potential etiological agent in an SFP outbreak and need to be taken in account for risk assessment. Moreover, the effect of the environment on enterotoxin expression should be considered when assessing the risk of SFP. Various studies evidence the limitation on the reliability of risk assessment based on the assumption that cell growth and enterotoxin production are related (Marta et al., 2011; Zeaki et al., 2014; Schelin et al., 2017). In addition to what was mentioned above for the probability of toxin production it is also the *S. aureus* growth rate that follows different patterns from those of enterotoxin production, depending on growth conditions and strain. For SEA, more studies, including different *S. aureus* food isolates carrying different *Siphoviridae* bacteriophages could further improve our knowledge concerning the regulation of expression. The schematic representation of the events regulating prophage induction in the study by Zeaki et al. (2015a) could serve as a template into which more information on *sea* gene expression could be incorporated to create a model including the factors that should be considered during risk assessment for SFP (Figure 2). Furthermore, additional data on the growth of *S. aureus* and SEA production on various food matrices could improve quantitative models for more accurate estimates of SEA production.

Another challenge that remains to be overcome when assessing the risk of *S. aureus* intoxication is the number of *se* genes carried by one strain. As shown by relevant studies and SFP outbreaks, it is quite common for more than one *se* gene to be detected in *S. aureus* strains implicated in intoxication cases (see e.g., Hennekinne et al., 2009 [*sea, sed, sej*]; Kitamoto et al., 2009 [*sea, sed*]; Schmid et al., 2009 [*sea, sed*]; and Johler et al., 2015 [*sea, sed*] in Table 1). One of the most recent examples is an outbreak documented by Johler et al. (2015), which occurred in a Swiss boarding school and affected 14 individuals. The *S. aureus* strain identified as the source of that outbreak harbored both *sea* and *sed*. Likewise, both SEA and SED were recovered from the implicated food (soft cheese) at levels of >6 ng/g cheese and >200 ng/g cheese, respectively. Since SEA and SED production are regulated by different mechanisms, it is evident that more information is needed on how these mechanisms are triggered in different food environments.

Identifying the point of entry of the *S. aureus* strain into the food chain and the clonal lineage to which it belongs will provide information that could be used in risk assessment profiles for identification of relevant preventive controls. A recent study by Kümmel et al. (2016) provides insight in this matter. Through the collection of samples from cow to retail product (ripened semi-hard cheese) at 18 dairy farms, the authors found that the bovine udder constitutes an important source of *S. aureus* in the dairy industry. Strains of genotype B were those most successfully transmitted from cows into the dairy production chain. The strain implicated in the outbreak at the Swiss boarding school was also assigned to an *S. aureus* of genotype B (Johler et al., 2015). Similarly, the study by Hummerjohann et al. (2014) showed that genotype B strains are primarily found in semi-hard cheeses produced from raw milk.

It should be borne in mind that pathogenic bacteria evolve with consumers’ changing habits and the development of food production methods, and that microbial risk assessment should also develop in a similar manner. A typical example of studies in this direction is the one assessing the impact of temperature fluctuations in toxin production of *S. aureus* in deli meals (Røssvoll et al., 2014). Moreover, the recognition that bacterial responses are the result of a complex network of genetic events should direct research efforts toward a better understanding of these mechanisms, with the aim of more efficient pathogen control throughout the food production chain.

## Concluding Remarks

*Staphylococcus aureus* is a highly evolved pathogen that brings about a number of challenges to food production. The physiological properties of the bacterium as such pose the first challenge for SFP containment. *S. aureus* robustness for growth along with its natural niches assists in the establishment of the pathogen in a broad range of food products. Poor hygiene habits by food handlers further facilitate its entrance into the food chain. In addition, *S. aureus* is an evolutionary flexible pathogen. Horizontal gene transfer is one of the main ways this bacterium acquires virulence factors. Therefore, the continual changes in food habits and processing methods trigger the adaptation mechanisms of the pathogen. The latter has brought about new strains exhibiting increased virulence and resistance to the applicable preservation methods. The variety of SEs produced by *S. aureus* and the complexity of their regulation and production must moreover be carefully considered. Food parameters can critically affect SE production and hence the virulence of *S. aureus*. Understanding the mechanisms behind the expression of an SE and how they can be influenced by for example the food composition, salt content, and pH constitutes the greatest of the challenge to overcome in the battle against SFP. To conclude, more targeted studies of different SE regulatory mechanisms in food will be the key to allow for improved understanding of SFP and to enable risk assessment. Finally, considering the exponential advances in the area of -omics disciplines, an ultimate challenge and emerging trend in food science is to integrate the molecular data underpinning the phenotypic responses of bacterial pathogens into predictive models and then into QMRA. This will enable our knowledge on the physiological response of pathogens, which may explain uncertain areas of their behavior (at least currently) to be expressed in quantitative terms and translated in explicit (numerical) terms of risk for used in proper decisions to guard food safety.

## Author Contributions

NZ designed this review, performed the literature study, prepared Table 1 and Figure 1, 2, and wrote the major part of the manuscript. SJ and JS prepared Table 2. SJ, PS, and JS contributed in writing and critical revision. All authors approved the final version of the manuscript. JS was the principal supervisor and responsible for manuscript preparation.

## Conflict of Interest Statement

The authors declare that the research was conducted in the absence of any commercial or financial relationships that could be construed as a potential conflict of interest.
